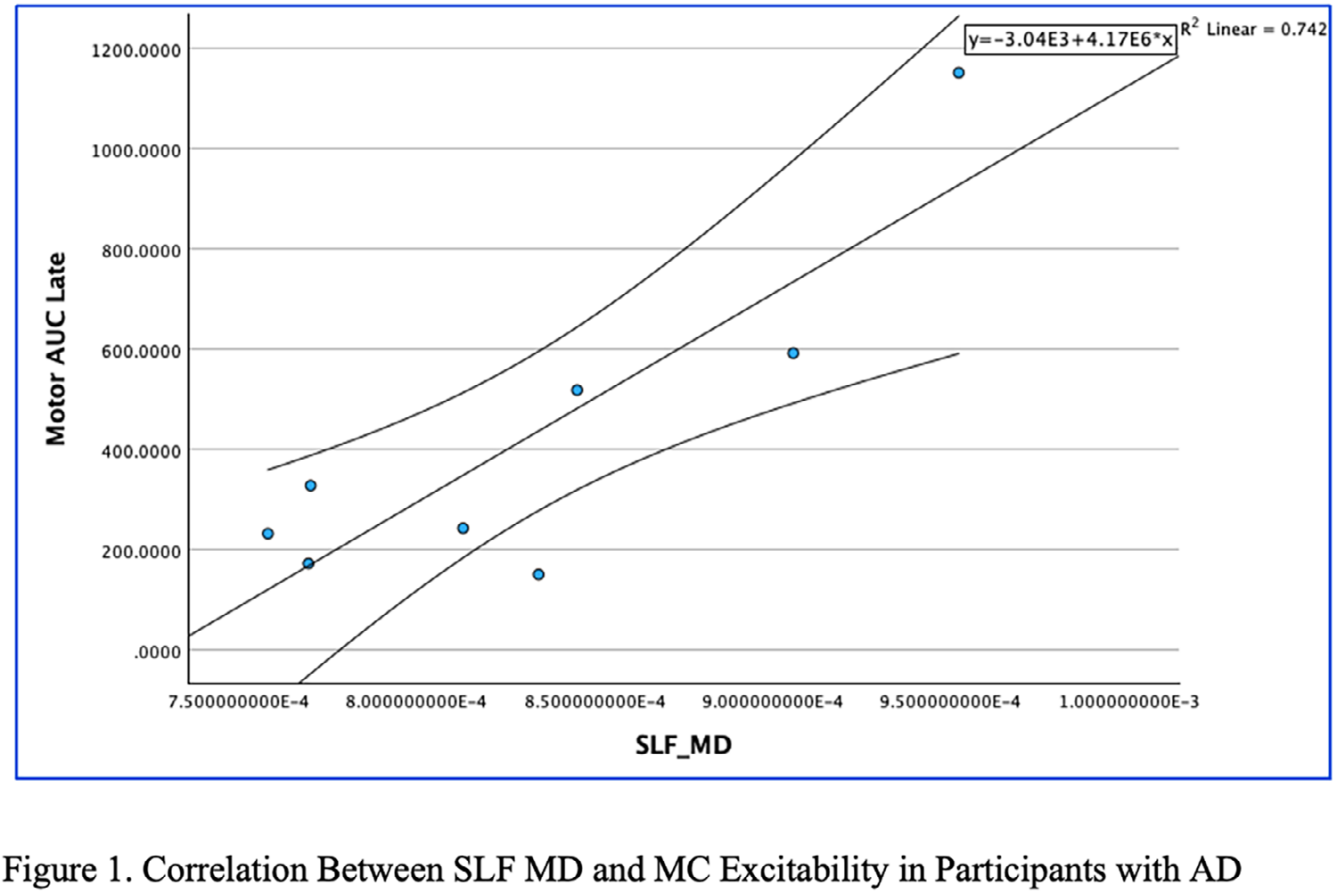# Assessing hyperexcitability in the context of cortical gray matter structures and white matter integrity in the dorsolateral prefrontal cortex and motor cortex of Alzheimer’s dementia patients

**DOI:** 10.1002/alz70862_109850

**Published:** 2025-12-23

**Authors:** Jessica Hira, Reza Zomorrodi, Daniel M. Blumberger, Angela C Golas, Benoit H. Mulsant, Bruce G. Pollock, Tarek K Rajji, Aristotle N. Voineskos, Sanjeev Kumar

**Affiliations:** ^1^ Temerty Faculty of Medicine, University of Toronto, Toronto, ON Canada; ^2^ Temerty Centre for Therapeutic Brain Intervention, CAMH, Toronto, ON Canada; ^3^ Temerty Centre for Therapeutic Brain Intervention CAMH, Toronto, ON Canada; ^4^ Centre for Addiction and Mental Health, Toronto, ON Canada; ^5^ Department of Psychiatry, Temerty Faculty of Medicine, University of Toronto, Toronto, ON Canada; ^6^ Campbell Family Mental Health Research Institute, Centre for Addiction and Mental Health, Toronto, ON Canada; ^7^ UT Southwestern University, Dallas, TX USA; ^8^ Adult Neurodevelopment and Geriatric Psychiatry Division, CAMH, Toronto, ON Canada

## Abstract

**Background:**

Abnormal cortical excitability is a marker of neurodegeneration in Alzheimer’s dementia (AD). However, the link between cortical excitability and structural changes in AD is not well understood. The objective of this study is to assess the relationship among motor cortex (MC) excitability, cortical thickness, and white matter integrity. We hypothesized that there is an inverse association between MC excitability and thickness or white matter tract integrity assessed from superior longitudinal fasciculus (SLF).

**Method:**

Participants were older individuals with AD meeting core National Institute on Aging and Alzheimer's Association (NIA‐AA) clinical criteria or cognitively normal (CN) older individuals. Single‐pulse TMS was delivered to the MC using a 7‐ cm figure‐of‐eight coil and Magtism 200 stimulator. EEG was recorded during the TMS protocol using a 64‐channel Synamps 2 EEG system with DC at 20 kHz sampling rate. A rectified area under the curve between 50‐275 ms post‐TMS‐evoked potential was used to measure excitability. T1‐weighted MRI scans were pre‐processed using established pipelines and estimates of cortical thickness were generated using FreeSurfer v6.0.1. Mean diffusivity (MD) and fractional anisotropy (FA) of the SLF were measured from diffusion‐weighted MRI data using the ENIGMA‐DTI protocol.

**Result:**

60 participants with AD (39 females, mean age = 74.0, *SD* = 8.8) and 40 CN participants (27 females, mean age = 67.1, *SD* = 7.8) were included. Participants with AD had reduced MC thickness (t_74_ = ‐3.414, *p* = 0.001), increased SLF MD (t_60_ = 2.364, *p* = 0.021), and decreased FA (t_60_ = ‐2.437, *p* = 0.018). In 31 participants with both MRI and TMS‐EEG data, MC excitability did not differ between AD and CN groups. In the AD group, SLF MD correlated positively with MC excitability (*r* = 0.861, df = 7, *p* = 0.006). No relationships were found between MC excitability and cortical thickness or FA.

**Conclusion:**

The SLF is a major association pathway that interconnects the frontal lobe with other brain regions and is implicated in motor control. The positive correlation between SLF MD and MC excitability in AD may be related to a compensatory response of the MC in response to neurodegeneration.